# Game Changing Solutions in Healthcare

**DOI:** 10.12669/pjms.40.2(ICON).9128

**Published:** 2024-01

**Authors:** Ammad Fahim, Sayyeda Ezra Reza

**Affiliations:** 1 Ammad Fahim, MBBS, MPhil, PhD. Office of Research, Innovation & Commercialization (ORIC), Indus Hospital & Health Network Karachi, Pakistan; 2 Sayyeda Ezra Reza, Office of Research, Innovation & Commercialization (ORIC), Indus Hospital & Health Network Karachi, Pakistan

With recent innovations and technological advancements, progressions in healthcare are occurring at an astonishing rate. The catastrophe of the recent pandemic also challenged our existing infrastructure propelling us to re-look into our health systems for reformation. When Covid 19 in 2020 completely changed the way, we interacted personally and professionally, the health sector was forced to find analogues means to serve and preserve. And looking back, we see how this austerity made a many of us a promoter of the ‘avant-garde’ for survival.

In this rapidly evolving world, healthcare providers are in quest of solutions that can save energy and time while being cost viable with best results. This calls forth a need for awareness and understanding of the right technology with timely implementation; a necessity for every healthcare institution to derive better health outcomes.

Mobile phones are an integral companion today, hence, utility of mobile apps for following vitals, heart rate, BP, oxygen saturation is not only increasing but has now holistically brought forth an entire mHealth- ecosystem into picture. Use of artificial intelligence (AI) in providing healthcare solutions has now edged into a reality from a mere trend. AI has contributed to considerable cut-backs in health care trajectories such as minimum time consumption in preclinical trials with significant cost reduction in drug discovery, and lesser workload and lab turnaround time in diagnostic automation; improving patient care. Machine learning algorithms deployed in AI provide predictive analytics for decision support, potential complications and patient outcomes thus aiding in personalized medical care. The advent of gene editing technologies like CRISPR/Cas9 has revolutionized gene modification, marking its significant utility in pharmaceutical industry for 1^st^ generation CRISPR/Cas9 therapeutics for monogenic diseases like Thalassemia, Sickle cell disease, Cystic Fibrosis and in cell therapies such as in cancer immunotherapy.^1^-^3^ Research on the human microbiome constituting all microbes in a person residing symbiotically has been directly linked to human health and wellness. There has been very interesting preclinical and clinical evidence of gut microbiome as key susceptibility determinant in Parkinson’s Disease, Stroke, Alzheimer’s Disease, Multiple Sclerosis and Autism Spectrum Disorder.^4^

Motivated with the invigoration and inspiration of the said scientific advances, we captured sentiments and efforts surrounding this change in work ethics in our 7^th^ Biennial ICON 2024, themed ‘Game Changing Solutions in Healthcare’ to showcase Indus’s innovative endeavors in healthcare research. A sincere gratitude to authors who responded enthusiastically to our call for publication- a thank you is just a way to begin appreciating your interest and efforts. We received 52 submissions for publications from over 22 specialties, as 35 original articles, 12 case reports and three editorials. Eventually fourteen original articles, one short communication, six case reports, and two correspondence were accepted for publication. Our call was met with a robust response from all campuses. This year’s conference has a unique edge to it – participation of both The North and The South campuses for the first time and once again gathering for a face-to-face conference. We foresee a rich enthusiastic amalgamation of people of Indus Network to celebrate and learn from one another with a widened scope and collaboration; beginning with the conference Opening at Jubilee campus Lahore, and Closing of the event at Karachi.

Indus’s realization for game changing solutions, is a reflection of our drive for innovation in service for humanity. Change as it is, is a complex entity; and a review of articles showed how, as Researchers, we begun to see impacting strategies in little things around us. Game changing solutions which echo of big bombastic strategies, very often isn’t so. Our write-ups highlighted perspectives of not just new ideas floating into the system but close examination of past cases, giving us new hope and new standpoints; unseen before.
